# 

**Published:** 1899-02

**Authors:** 


Vol. LIII. FEBRUARY, 1899 No. 2.

### THE DENTAL REGISTER

A MONTHLY JOURNAL.

Devoted to the Interests of the Profession.

EDITED BY

J. TAFT, D.D.S.

LIBRARY
SURGEON GENERAL'S OFFICE
FEB. -27. -1899

PUBLISHED BY

SAM'L A. CROCKER & CO.,
OHIO DENTAL AND SURGICAL DEPOT,
35, 37 and 39 West Fifth Street, CINCINNATI, OHIO.

Entered at the Postoffice at Cincinnati, O., as second class mail matter.

TERMS: $2.00 PER ANNUM IN ADVANCE. SINGLE COPIES, 25 CTS.

